# SHP2 knockdown ameliorates liver insulin resistance by activating IRS‐2 phosphorylation through the AKT and ERK1/2 signaling pathways

**DOI:** 10.1002/2211-5463.12992

**Published:** 2020-11-03

**Authors:** Xinxin Yue, Tao Han, Wei Hao, Min Wang, Yang Fu

**Affiliations:** ^1^ Department of Clinic College He University Shenyang China; ^2^ Department of Oncology The First Affiliated Hospital of China Medical University Shenyang China; ^3^ Department of Burn and Plastic Surgery General Hospital of Northern Theater Command Shenyang China

**Keywords:** diabetes, glucose consumption, insulin resistance, SHP2

## Abstract

Diabetes is a chronic metabolic disease characterized by insulin resistance (IR). SHP2 has previously been identified as a potential target to reduce IR in diabetes. Here, we examined the effects of SHP2 on glucose consumption (GC), IR level and the expression of insulin receptor substrate (IRS), AKT and extracellular signal‐regulated kinase (ERK)1/2 proteins in a cellular and animal model of diabetes. IR was induced in hepatocellular carcinoma (HCC) cells, and SHP2 was up‐regulated or down‐regulated in cells. Diabetic rats were treated with SHP2 inhibitor. GC of cells, and the weight, total cholesterol, triglycerides, fasting blood glucose, fasting insulin, homeostasis model assessment‐IR index and insulin sensitivity (ISI) of the rats were analyzed. The levels of SHP2 and the activation of IRS‐2, AKT and ERK1/2 in cells and rats were measured by quantitative real‐time PCR (qRT‐PCR) or western blot. GC was reduced, but expression of SHP2 was enhanced in IR HCC cells. Phosphorylation of IRS‐2 and AKT in IR HCC cells and diabetic rats was decreased, whereas phosphorylation of ERK1/2 was enhanced. In both the cell and animal models, SHP2 knockdown enhanced GC, ameliorated IR, activated IRS‐2 and AKT, and inhibited ERK1/2 phosphorylation, in contrast with the effects of SHP2 overexpression. SHP2 knockdown may enhance GC and ameliorate IR through phosphorylation of IRS‐2 via regulating AKT and ERK1/2 in liver.

## Graphical abstract

Here, we report that SHP2 knockdown enhances glucose consumption, ameliorates insulin resistance, activates IRS‐2 and AKT, and inhibits ERK1/2 phosphorylation in liver.

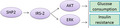

**Abbreviations**
FBG: fasting blood glucose
FINS: fasting insulin
GC: glucose consumption
HCC: hepatocellular carcinoma
HFD: high‐fat diet group
HOMA‐IR: homeostasis model assessment‐insulin resistance index
IR: insulin resistance
IRS: insulin receptor substrate
ISI: insulin sensitivity
NC: negative control
p: phosphorylated
qRT‐PCR: quantitative real‐time PCR
SD: standard deviation
siNC, silencing negative control; TC: total cholesterol
TG: triglyceride
WB: western blot

The incidence of diabetes is steadily increasing [1]. Diabetes is a chronic metabolic disease characterized by hyperglycemia, hyperinsulinemia and insulin resistance (IR), and the relationship between diabetes and various system tumors has been widely observed in clinical studies [2]. Previous findings have shown that diabetes increases the risk for development of cancers, such as breast [3], colorectal [4], pancreatic [5] and endometrial cancers [6]. In addition, increasing evidence supports the correlation between diabetes and liver cancer, and indicates that hyperinsulinemia, IR, oxidative stress and chronic inflammatory response are of concern with the possibility of patients with type 2 diabetes experiencing development of liver cancer [7, 8, 9]. According to the statistics, the incidence of liver cancer in patients with diabetes is obviously higher than in nondiabetic patients [10]. Moreover, the pathogenesis of diabetes‐related liver cancer was still unknown.

SHP2, a widely expressed nonreceptor tyrosine phosphatase, is encoded by *PTPNll* gene in humans, contains two SH2 domains and is verified to be involved in numerous cellular signal transduction pathways [11]. It has been shown that SHP2 is closely related to the occurrence of multiple solid tumors [12]. Moreover, SHP2 has been identified as an indispensable component in promoting the development and progression of liver tumors [13]. Han *et al*. [14] indicated that the suppression of SHP2 reduces the migration and adhesion of hepatocellular carcinoma (HCC) cells and the formation of HCC metastasis in a nude mouse xenograft assay. In the mechanism of action, previous findings showed that SHP2 mediated phosphoinositide 3‐kinase/AKT, RAS/extracellular signal‐regulated kinase (ERK) and other signaling pathways to affect the growth, invasion and migration of tumor cells [15]. In addition, SHP2 has already been confirmed as a target for diabetes to decrease IR [16].

This study explored the role and its relevant mechanism of SHP2 in diabetes‐related organ liver by conducting cell and animal experiments. Diabetes was induced by IR in HCC cells, and SHP2 was up‐regulated or down‐regulated in the cells, while diabetic model rats were treated with SHP2 inhibitor. Furthermore, we determined the effects of SHP2 on glucose consumption (GC), IR level and the expressions of insulin receptor substrate (IRS), AKT and ERK1/2 proteins both in cells and in rats. The purpose of this study was to clarify the function of SHP2 in the diabetic liver of diabetes, hoping to provide a potential treatment for the disease.

## Materials and methods

### Ethics statement

All animal experiments were approved by the Ethic Committee of Liaoning Ho’s Medical College. The experimental operations were performed strictly following the guidelines of Animal Care and Institutional Ethical Commitee in China.

### Cell culture and induction

Human HCC cells Huh7 were purchased from Chinese Academy of Typical Culture Collection Cell Bank (Shanghai, China) and used to construct IR cell models. All of the cells were incubated in Dulbecco's modified Eagle's medium (DMEM; Gibco, New York, NY, USA) containing 10% FBS (Gibco), 100 U·mL^−1^ penicillin and 0.1 mg·mL^−1^ streptomycin (Gibco). Huh7 cells were maintained in an incubator filled with 5% CO_2_ at 37 °C, and only cells in logarithmic growth were used in subsequent experiments. The IR cell model was constructed as previously described [17]. Huh7 cells (1 × 10^4^ cells·mL^−1^) were seeded into 96‐well plates and induced in a high‐sugar DMEM culture medium containing 1 μm dexamethasone (Sigma‐Aldrich, St. Louis, MO, USA) and 10% FBS for 48 h. For comparison, cells with induction (IR) served as controls.

### Cell transfection

To investigate the effect of SHP2 on the IR cell models, we transfected the Huh7 cells with empty vector as negative control (NC), silencing negative control (siNC), SHP2‐pcDNA3.1 or siSHP2, and the transfection efficiency was detected by RT‐PCR. Besides, cells were transfected with siSHP2 plus NC, siNC plus SHP2‐pcDNA3.1 or NC plus siNC; then the transfected cells (1 × 10^4^ cells·mL^−1^) were seeded into 96‐well plates and induced in a high‐sugar DMEM culture medium containing 1 μm dexamethasone and 10% FBS for 48 h for subsequent assays. The transfection was performed using Lipofectamine 2000 transfection reagent (Invitrogen, Carlsbad, CA, USA) according to the instructions. The base sequences involved in this study were synthesized by Gene Pharma (Suzhou, China).

### Glucose content detection

The GC in the medium of different groups was then determined using a glucose assay kit (#F006‐1‐1; Nanjing Jiancheng Bioengineering Institute, Nanjing, China) according to the manufacturer's instructions: GC = the initial glucose level − the remaining glucose level in the medium.

### Diabetic rat model

A total of 60 male Sprague‐Dawley rats (6 weeks old, weighing 180–220 g) were purchased from the Guangdong Medical Laboratory Animal Center (Guangdong, China). All of the rats were raised under a sterile condition at the temperature of (21 ± 2 °C) with the relative humidity of 50–70% and 12‐h dark/light cycle, and were allowed to have access to food and water freely. After 1 week of adaptive feeding, the rats were divided into normal diet group (normal, *n* = 10), high‐fat diet group (HFD; *n* = 25) and HFD + SHP099 group (*n* = 25). The HFD was composed of 10% commission powder, 10% saccharose, 20% lard and 60% normal diet. The rats in the HFD + SHP099 group were orally administrated with SHP2 inhibitor (SHP099 solution, 30 mg·kg^−1^; #HY‐100388; MedChemExpress, Monmouth Junction, NJ, USA) on the basis of HFD. The rats were all fed for 9 weeks, and their weight was recorded once a week.

### Blood detection

After 9 weeks of feeding, all of the rats were fasted for 8 h. The blood samples of rats were then collected through the posterior orbital venous plexus under ether anesthesia. The serum was obtained by centrifuging the blood samples at 3500 ***g*** for 10 min. The serum level of rat lipids, including total cholesterol (TC) and triglycerides (TGs), was detected by an automatic analyzer (AU2700; Olympus, Tokyo, Japan). The fasting blood glucose (FBG) content in the rat serum was assessed by the CareSens blood glucose meter and corresponding paper (i‐SENS, Seocho‐gu, Seoul, Korea). The level of fasting insulin (FINS) was measured by the ELISA according to the kit instructions (#XF02128‐96T; Shanghai Xinfan Biotechnology Co., Ltd, Shanghai, China). Furthermore, the homeostasis model assessment‐insulin resistance index (HOMA‐IR) and insulin sensitivity (ISI) were calculated: HOMA‐IR = (FBG × FINS)/22.5 and ISI = ln [1/(FBG × FINS)].

### Tissue samples

At the end of the experiment, the rats were sacrificed by intraperitoneal injection of pentobarbital sodium (150 mg·kg^−1^) to collect their liver tissues. The tissue samples were then fixed in 4% paraformaldehyde for 24 h, sliced into 5‐µm sections, paraffin embedded and reserved at −80 °C for further detection.

### Quantitative real‐time PCR assay

The expression of SHP2 in the treated Huh7 cells and rats was determined by quantitative real‐time PCR (qRT‐PCR) assay. In detail, the total RNAs of cells and tissues were separated by TRIzol reagent (Invitrogen), and the quality and integrity of the separated RNAs were respectively evaluated by the NanoDrop‐2000c spectrophotometer (Thermo Fisher Scientific, Waltham, MA, USA) and 1% agarose modified gel electrophoresis. The first‐strand cDNAs were synthesized from the isolated RNA (1 µg) with the PrimeScript RT Master Mix Perfect Real Time (TaKaRa, Shiga, Japan) following the instructions. qRT‐PCR assay was conducted in the ABI Prism 7500 Fast Real‐time PCR System (Applied Biosystems, Foster City, CA, USA) under the following reaction conditions: 3 min at 94 °C, 40 cycles of 30 s at 50 °C and 50 s at 72 °C, followed by 8 min at 72 °C. The mRNA expression was determined by the comparative $2^{‐\Delta \Delta C_{t}}$ method [18], and GAPDH was used as an internal reference. The sequences of primers were purchased from Gene Pharma and are listed in Table 1.

**Table 1:** Primer base sequence.

Gene	Forward (5′–3′)	Reverse (5′–3′)
*SHP2*		
Human	ACGGCAAGTCTAAAGTGA	AGGTCCGAAAGGTGGTATT
Rat	GCAGTGCTGGGATTGGCCGGACAGGAAC	CAGTCCAACCCCGGCGAGCTTCTGAACAC
*GAPDH*		
Human	ACCACAGTCCATGCCATCAC	TCCACCACCCTGTTGCTGTA
Rat	GCATGGCCTTCCGTGTTCCTA	AGTGTTGGGGGCTGAGTTGG

### Western blot analysis

The expression levels of SHP2, IRS‐2, Akt and ERK in the treated Huh7 cells and rats were analyzed by western blot (WB). In brief, the total proteins of cells and tissues were lysed using radioimmunoprecipitation assay buffer (Beyotime Institute of Biotechnology, Jiangsu, China), and the protein concentration was measured by Bicinchoninic Protein Assay kit (Pierce, Rockford, IL, USA). Forty micrograms protein samples was separated on 10% SDS/PAGE (Beyotime, Shanghai, China) and then electrotransferred onto polyvinylidene fluoride membranes (Millipore, Billerica, MA, USA). Next, the membranes were blocked with 5% nonfat dry milk at room temperature for 1 h and then exposed to the following primary antibodies (Abcam, Cambridge, MA, USA) overnight at 4 °C: SHP2 (1 : 1000, ab131541), phosphorylated (p)‐IRS‐2 (1: 3000, ab3690), IRS‐2 (1 : 2000, ab134101), p‐AKT (1 : 1000, ab38449), AKT (1 : 10 000, ab179463), p‐ERK1/2 (1 : 1000, ab214362), ERK1/2 (1 : 1000, ab17942) and GAPDH (1 : 1000, ab181602), which was an internal reference. Subsequently, the homologous secondary antibody goat anti‐(rabbit IgG H&L) (HRP; 1 : 7000, ab97051) was added at room temperature for another hour. The blots were developed with the enhanced chemiluminescence‐detecting kit (ECL; Thermo Fisher).

### Statistical analysis

statistical package of the social sciences 20.0 software (SPSS, Inc., Chicago, IL, USA) was used for data analysis. The data were presented as mean ± standard deviation (SD). The difference between groups was compared by one‐way ANOVA followed by Tukey's *t*‐test. All experiments were performed in triplicate. A *P* value <0.05 was considered statistically significant.

## Results

### SHP2 expression was increased in IR HCC cell models

**Fig. 1:**
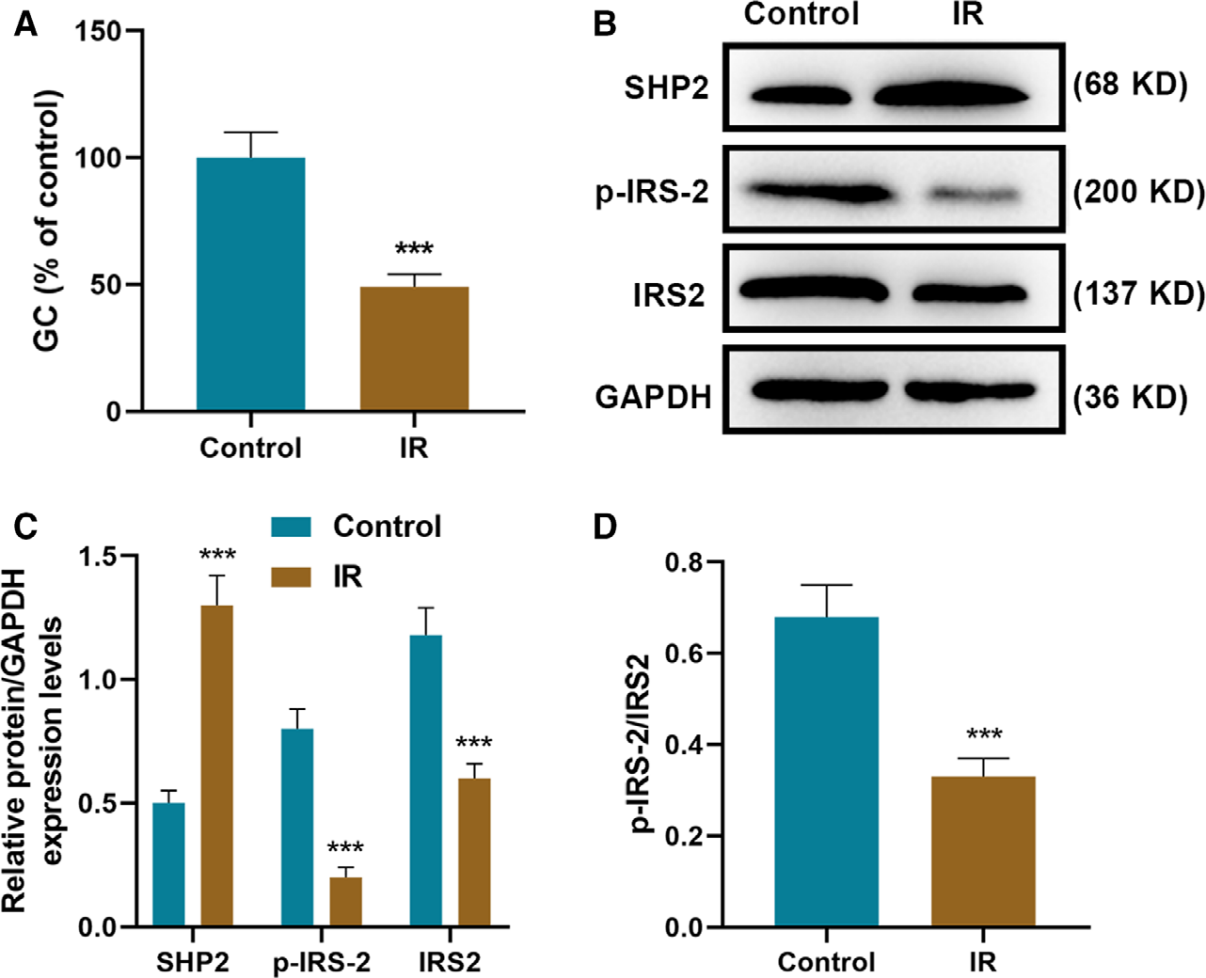
SHP2 expression was up‐regulated in IR HCC cell models. (A) The GC levels of IR HCC cell models and control cells were determined using a glucose assay kit. (B, C) The expressions of SHP2, p‐IRS‐2 and IRS‐2 in IR HCC cell models and control cells were analyzed by WB analysis, and (D) the ratio of p‐IRS‐2/IRS‐2 was further calculated. ***P < 0.001, versus control. *n* = 3. Data were presented as mean ± SD. Statistical differences were compared by ANOVA followed by Tukey's *t*‐test.

Compared with control cells, the GC of IR HCC cell models was observably reduced (*P* < 0.001; Fig. 1A). WB results revealed that the expression of SHP2 was obviously elevated in IR HCC cell models, whereas the expressions of p‐IRS‐2 and IRS2 were decreased (*P* < 0.001; Fig. 1B,C). Accordingly, the ratio of p‐IRS‐2/IRS2 was decreased visibly in IR HCC cell models in comparison with the control cells (*P* < 0.001; Fig. 1D).

### SHP2 knockdown alleviated IR by promoting phosphorylation of IRS‐2, Akt and ERK

**Fig. 2:**
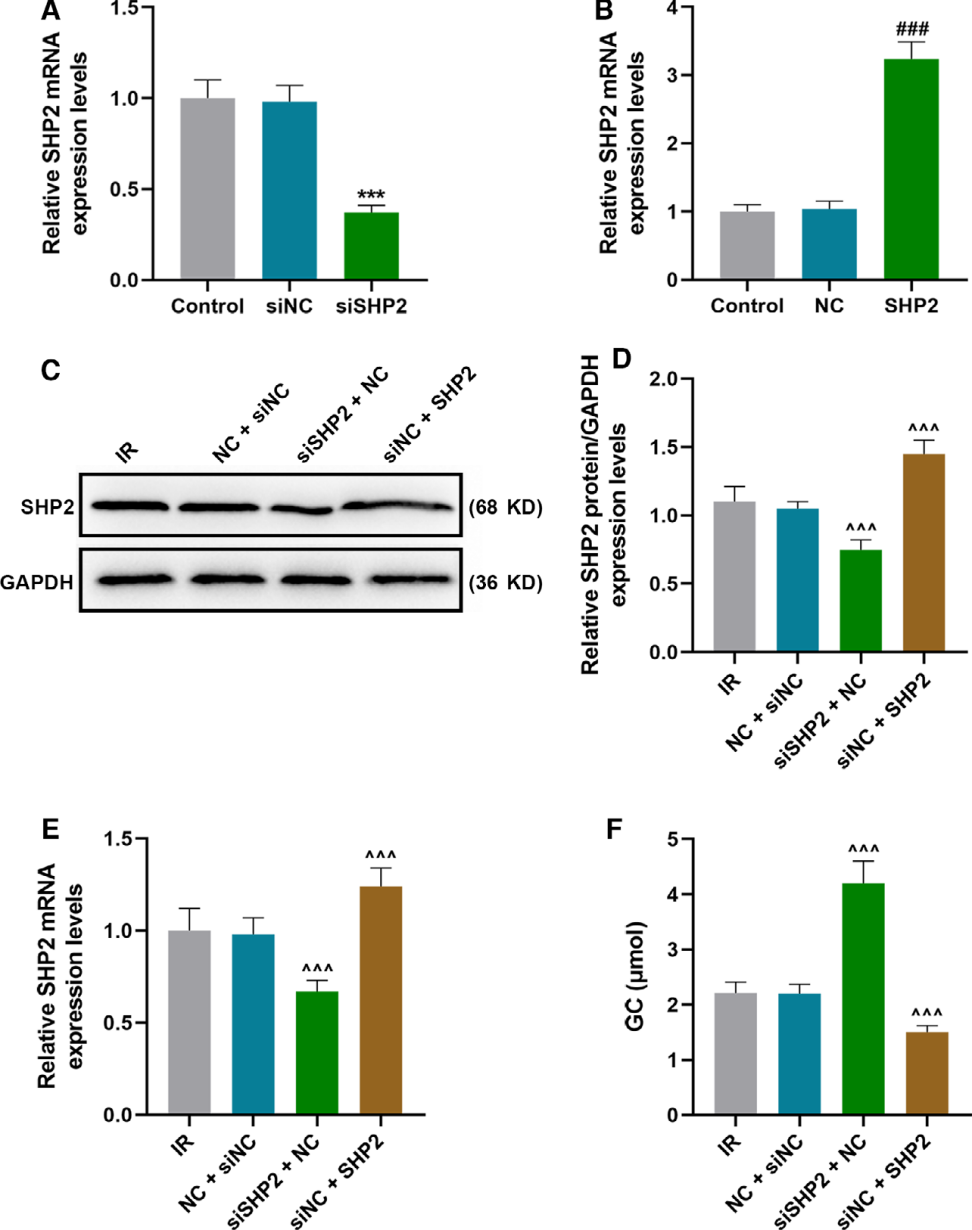
SHP2 knockdown alleviated IR in HCC cells. (A, B) The transfection efficiency of siSHP2 and SHP2 was detected by qRT‐PCR. (C–E) After IR treatment, the expression of SHP2 in transfected cells was analyzed by (C, D) WB analysis and (E) qRT‐PCR assay. (F) The GC level of transfected cells was determined using a glucose assay kit. ***P < 0.001, versus control. ^###^ *P* < 0.001, versus NC. ^^^^^ *P* < 0.001, versus NC + siNC. *n* = 3. Data were presented as mean ± SD. Statistical differences were compared by ANOVA followed by Tukey's *t*‐test.

**Fig. 3:**
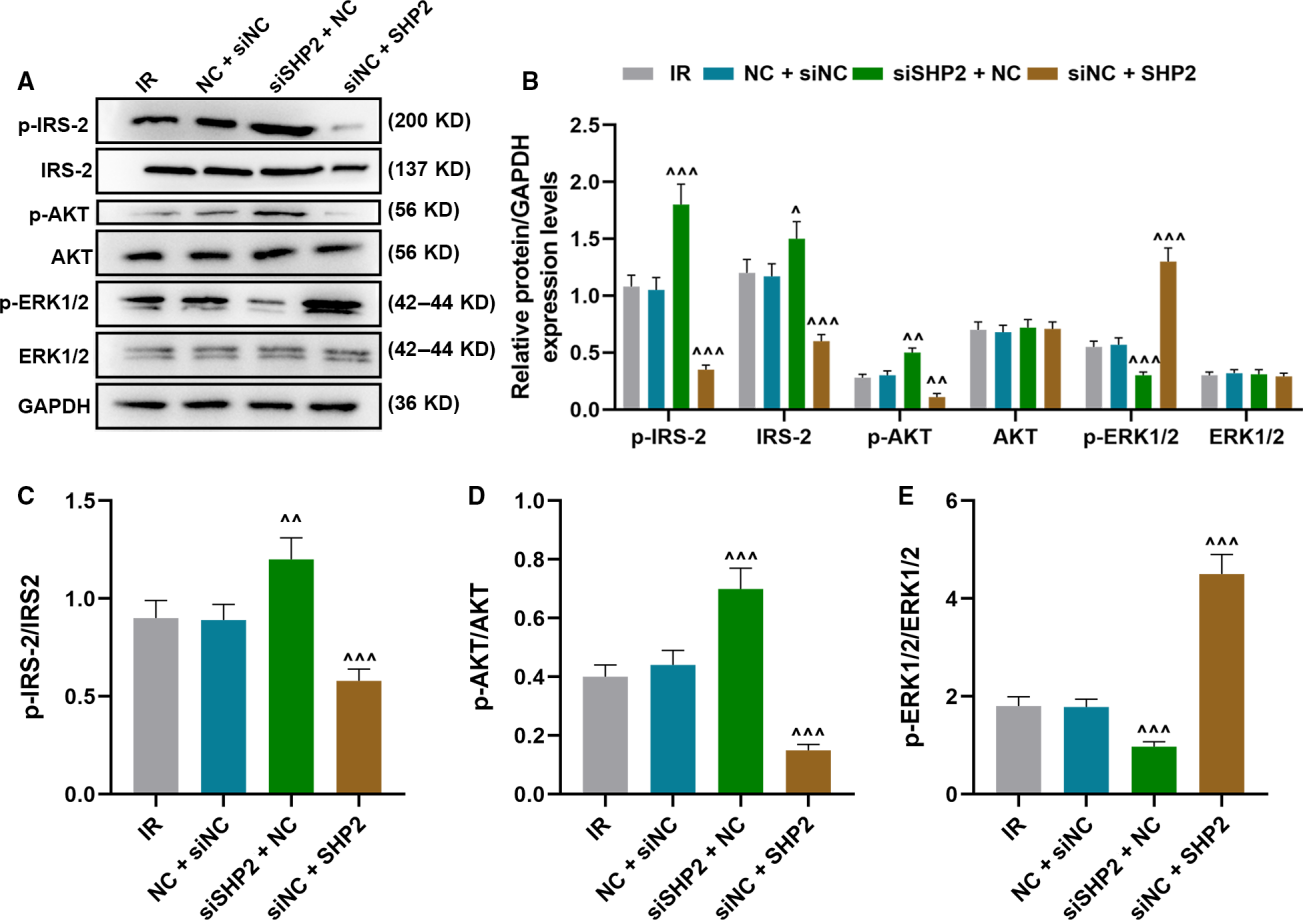
SHP2 knockdown alleviated IR by promoting phosphorylation of IRS‐2, Akt and ERK. In this figure, HCC cells were transfected with scrambled sequences (NC), siSHP2 plus NC or siNC plus SHP2 before IR induction. (A, B) The activations of IRS‐2, Akt and ERK in transfected cells were analyzed by WB analysis, and (C–E) the ratios of p‐IRS‐2/IRS‐2, p‐AKT/AKT and p‐ERK1/2/ERK1/2 were further calculated. ^^^ *P* < 0.05, ^^^^ *P* < 0.01, ^^^^^ *P* < 0.001, versus NC + siNC. *n* = 3. Data were presented as mean ± SD. Statistical differences were compared by ANOVA followed by Tukey's *t*‐test.

To explore the effect of SHP2 on the IR HCC cell models, this study up‐regulated and down‐regulated the expression of SHP2 in IR HCC cell models. First, the transfection efficiency of siSHP2 and SHP2 was detected by qRT‐PCR, and the expression of SHP2 in cells transfected with siSHP2 was significantly down‐regulated, but up‐regulated in cells transfected with SHP2 (*P* < 0.001; Fig. 2A,B). After IR treatment, compared with cells in the NC + siNC group, both qRT‐PCR and WB experiments verified that the expression of SHP2 in cells of the siSHP2 + NC group was significantly decreased but noticeably increased in the cells of the siNC + SHP2 group (*P* < 0.001; Fig. 2C–E). GC of IR HCC cell models was promoted by the down‐regulation of SHP2 but reduced by the overexpression of SHP2 (*P* < 0.001; Fig. 2F). In addition, as shown in Fig. 3, WB analysis indicated that SHP2 knockdown in IR HCC cell models increased the expressions of p‐IRS‐2, IRS‐2 and p‐AKT and reduced that of p‐ERK1/2 (*P* < 0.05). Correspondingly, the ratios of p‐IRS‐2/IRS‐2 and p‐AKT/AKT were increased noticeably under SHP2 knockdown, whereas the ratio of p‐ERK1/2/ERK1/2 was decreased greatly (*P* < 0.001). In addition, the effects of overexpressed SHP2 on the expressions of earlier proteins in IR HCC cell models were completely opposite to those of SHP2 knockdown.

### SHP2 inhibitor improved diabetic rats by promoting the phosphorylation of IRS‐2, Akt and ERK

**Fig. 4:**
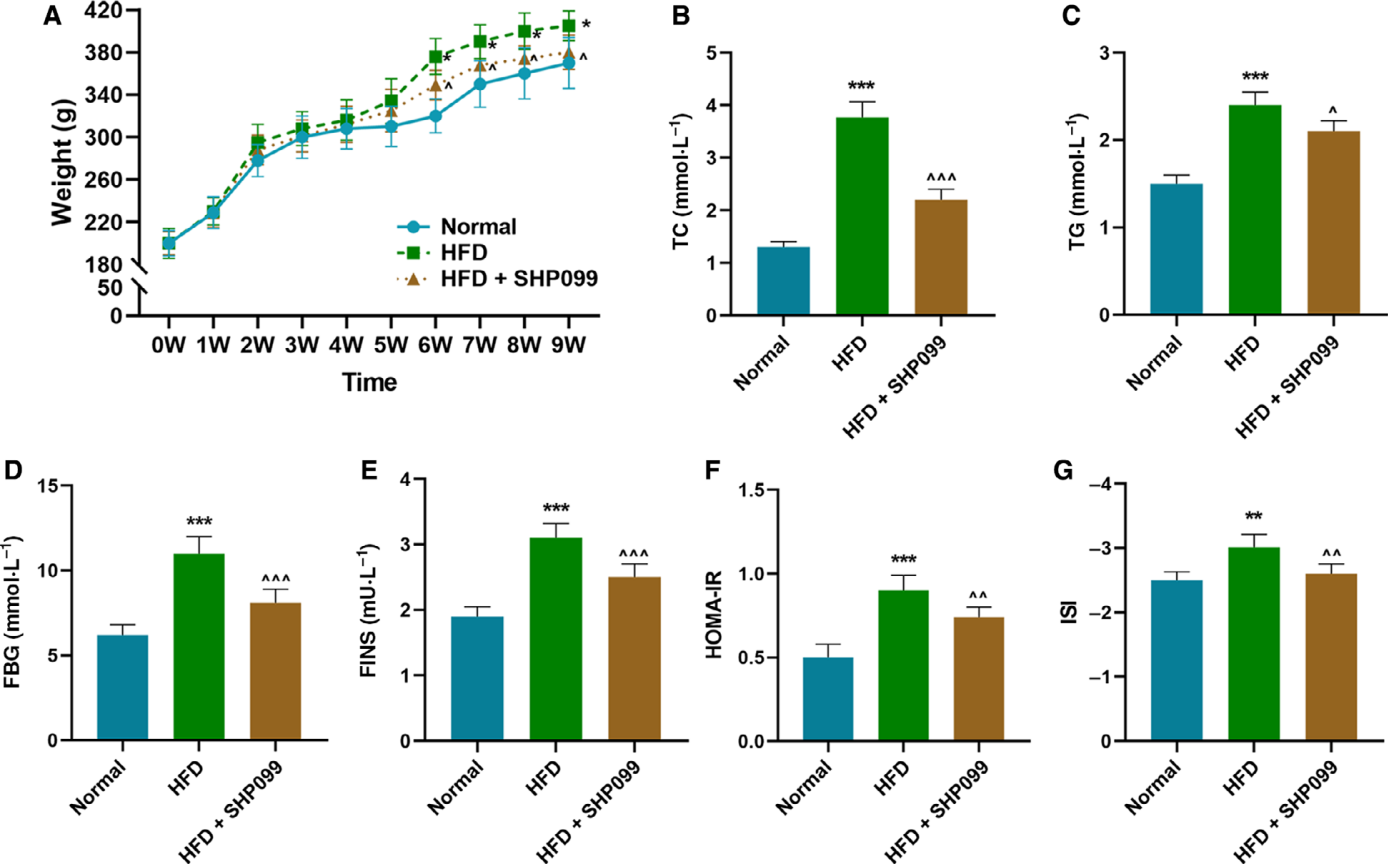
SHP2 inhibitor improved IR in diabetic rats. In this figure, a total of 60 rats were divided into normal diet group (normal, *n* = 10), HFD group (*n* = 25) and HFD + SHP099 group (*n* = 25). (A) Rats in each group were fed for 9 weeks, and their weight was recorded once a week. The levels of (B) TC, (C) TGs, (D) FBG, (E) FINS, (F) HOMA‐IR and (G) ISI of rats in different groups were detected and calculated, respectively. *P < 0.05, **P < 0.01, ***P < 0.001, versus normal; ^^^ *P* < 0.05, ^^^^ *P* < 0.01, ^^^^^ *P* < 0.001, versus HFD. *n* = 3. Data were presented as mean ± SD. Statistical differences were compared by ANOVA followed by Tukey's *t*‐test.

**Fig. 5:**
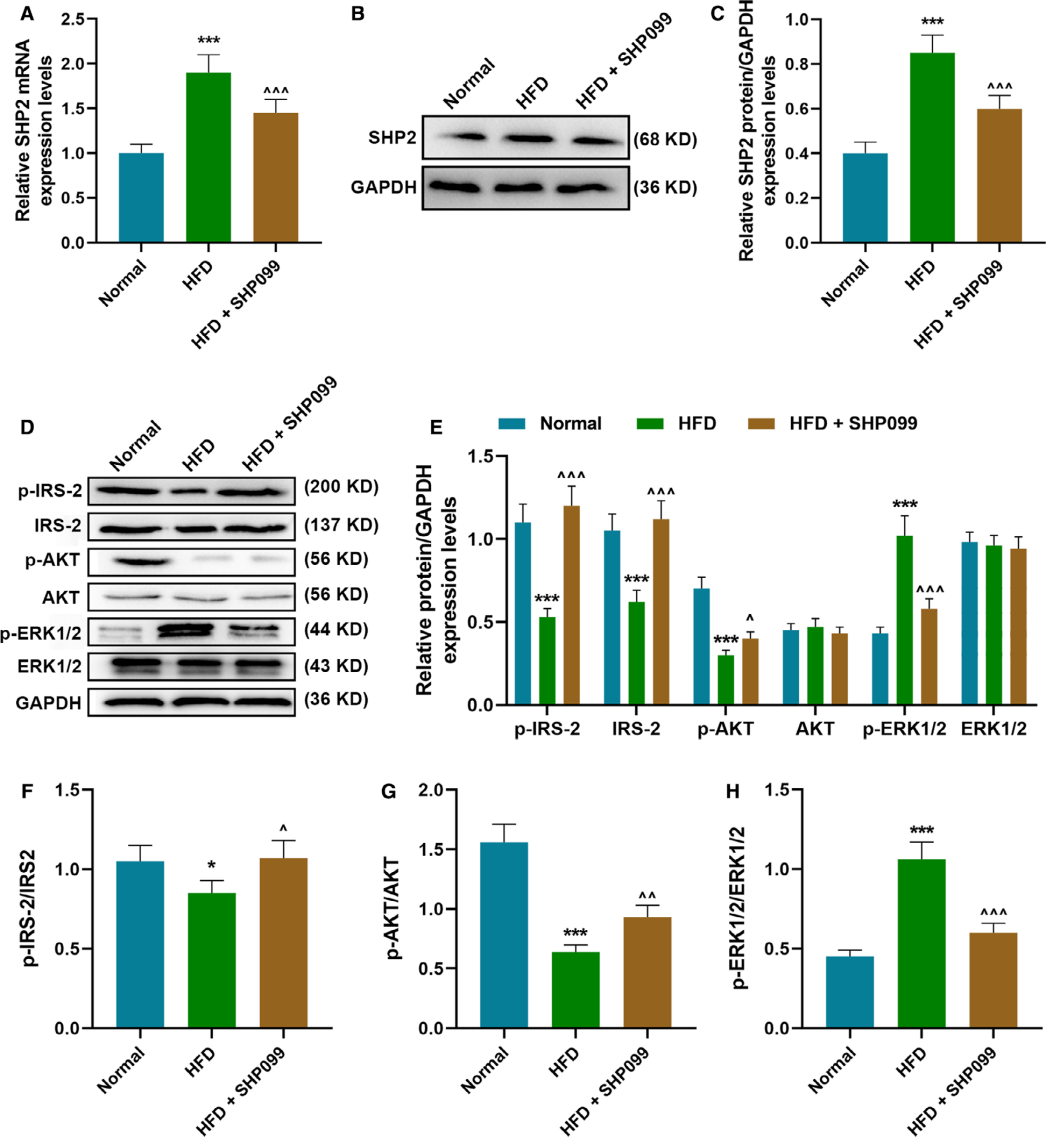
SHP2 inhibitor improved the diabetic rats by promoting phosphorylation of IRS‐2, Akt and ERK. In this figure, a total of 60 rats were divided into normal diet group (normal, *n* = 10), HFD group (*n* = 25) and HFD + SHP099 group (*n* = 25). The expression level of SHP2 in treated rats was analyzed by (A) qRT‐PCR assay and (B, C) WB analysis in liver tissues. (D, E) The activations of IRS‐2, Akt and ERK in liver tissues of rats were analyzed by WB analysis, and (F–H) the ratios of p‐IRS‐2/IRS‐2, p‐AKT/AKT and p‐ERK1/2/ERK1/2 were further calculated in liver tissues. *P < 0.05, ***P < 0.001, versus normal; ^^^ *P* < 0.05, ^^^^ *P* < 0.01, ^^^^^ *P* < 0.001, versus HFD. *n* = 3. Data were presented as mean ± SD. Statistical differences were compared by ANOVA followed by Tukey's *t*‐test.

To further investigate the role of SHP2 in diabetes, we constructed the diabetic rat models and treated them with a selective inhibitor of SHP2 (SHP099). Compared with normal diet, the body weight of diabetic rat models induced by HFD increased significantly, whereas SHP2 inhibitor mitigated overweight caused by HFD (*P* < 0.05; Fig. 4A). In blood detection, the experimental results demonstrated that the levels of TC, TGs, FBG, FINS and HOMA‐IR were increased and ISI was reduced noticeably in HFD‐induced rats, which, however, could be rescued by the intervention of SHP2 inhibitor (*P* < 0.05; Fig. 4B–G). In WB analysis, we observed that the expression of SHP2 was notably increased in HFD‐induced rats but was suppressed by SHP2 inhibitor (*P* < 0.001; Fig. 5A–C). Moreover, p‐IRS‐2 and p‐AKT were reduced and p‐ERK1/2 was evidently promoted in HFD‐induced rats, but these changes could be reversed by SHP2 inhibitor (*P* < 0.05; Fig. 5D,E). The ratios of p‐IRS‐2/IRS‐2 and p‐AKT/AKT were reduced, and that of p‐ERK1/2/ERK1/2 was elevated accordingly in HFD‐induced rats, which, however, could be reversed under the treatment of SHP2 inhibitor (*P* < 0.05; Fig. 5F–H).

## Discussion

A large amount of epidemiological evidence indicates that diabetes is distinctively correlated with the development of tumors, and patients with diabetes are more likely to experience liver cancer than those without [19]. At present, how diabetes affects liver cancer still remains unclear. Some evidence suggested that diabetes‐related liver cancer may be caused by IR, dyslipidemia and hyperglycemia [20, 21, 22]. In recent years, some studies found that SHP2 molecule and its mutants are closely involved in the disease courses of many malignancies [23]. Previously it has been reported that SHP2 was anomalously distributed and overexpressed in hematopoietic malignant tumors [24]. We simulated diabetes‐related liver cancer cell models by inducing IR to HCC cells and discovered that the expression of SHP2 was increased in cell models, suggesting that SHP2 could promote the occurrence of IR and thus trigger the progression of diabetes‐related liver cancer.

Liver is the main target organ of insulin action, and patients with diabetes‐related liver cancer show a high IR, which is characterized by the decreased sensitivity of cells and tissues to insulin, thus resulting in the decreased consumption of glucose [25]. At the molecular level, IR is mainly manifested as the blockage of intracellular signaling pathway after insulin binds to its receptors [26]. IRS is one of the most common substrates of insulin receptor, and its dysfunction and the decrease of expression will affect the transmission of insulin signaling, resulting in IR [27]. An animal study showed that IRS‐2 could rescue insulin signaling defects in mice, suggesting that IRS‐2 protein might be an important mediator of IR and related signaling pathway dysfunction [28]. Therefore, we speculated that improving IR and promoting IRS‐2 activation may have positive significance for diabetes‐related liver cancer. In this study, GC level and IRS‐2 phosphorylation in IR HCC cell models were suppressed greatly, indicating that establishing diabetes‐related liver cancer models in HCC cells by inducing IR to the cells was successful.

In liver diseases, SHP2 has been reported to promote hepatocyte regeneration after liver injury through regulating the AKT and ERK1/2 signaling pathways, and SHP2 knockout mice displayed very similar phenotypes of defective liver regeneration triggered by partial hepatectomy, including blunted ERK1/2 activation and AKT inhibition [29]. In addition, Chen *et al*. [30] also pointed out that the ERK1/2 and AKT signaling pathways were involved in the proliferation and metastasis of HCC cells. AKT and ERK, which belong to serine threonine kinases, play a key role in glucose metabolism, protein synthesis, and cell survival and proliferation [31, 32, 33]. AKT is a major downstream signaling pathway of insulin and can be activated after insulin binds to IRS to trigger glucose transforming protein and ultimately complete glucose uptake [34]. Activated ERK1/2 in a high‐glucose environment contributes to the development of diabetic complications [35]. Thus, AKT phosphorylation and ERK1/2 inhibition contribute to glucose uptake and IR. We determined that SHP2 knockdown effectively increased the GC in IR‐induced HCC cells, promoted the activation of IRS‐2 and AKT, and inhibited ERK1/2 phosphorylation. Thus, we speculated that SHP2 may stimulate the occurrence of diabetes‐related liver cancer, whereas SHP2 silencing could inhibit IRS‐2 phosphorylation by regulating the activation of AKT and ERK1/2, thus ameliorating glucose uptake and IR.

To further test our hypothesis, this study constructed *in vivo* diabetes rat models by feeding the rats with HFD and then analyzing the effect of SHP2 inhibitor on the rats. After feeding with HFD, the rat weight and levels of TC, TGs, FBG, FINS and HOMA‐IR in rats were increased significantly, but ISI value was decreased, indicating that the establishment of diabetes rat models was successful. In contrast, WB analysis demonstrated that the phosphorylation levels of IRS‐2 and AKT in HFD rats were obviously inhibited, but ERK1/2 was abnormally activated. Interestingly, all of these changes were reversed by the addition of SHP2 inhibitor, which was consistent with our previous cell experiment results suggesting that inhibiting SHP2 can improve glucose uptake disorder and IR in HFD rats via regulating the phosphorylation of IRS‐2, AKT and ERK1/2.

The study showed that the effect of SHP2 in the liver might contribute to IR. However, the oral administration of SHP2 inhibitor may involve the effects of tissues on facilitating restoration of TC, TGs and FBG to the near‐normal levels, and this was a limitation of this study.

The limitation of this study was not determining whether ERK inhibition alone also increases GC or whether SHP2 inhibition combined with AKT inhibition would abolish the effects on GC.

## Conclusions

In summary, our experimental finding verified that SHP2 expression was up‐regulated and IRS‐2 phosphorylation was significantly suppressed in the HCC cell‐based IR models. Furthermore, both *in vivo* and *in vivo* assays confirmed that SHP2 knockdown promoted GC and improved IR through activating IRS‐2 phosphorylation via the regulation of AKT and ERK1/2 signaling pathways. The study demonstrated that SHP2 in the diabetic mice could promote IR in the liver. The current findings improve the understanding of the role of SHP2 in diabetes and provide further experimental evidence for the treatment of the disease.

## Conflict of interest

The authors declare no conflict of interest.

## Author contributions

XY and TH substantially contributed to the conception and design. WH, MW and YF acquired the data, analyzed the data and interpreted the data. XY and TH drafted the article or critically revised it for important intellectual content. All authors approved the final version to be published and agreed to be accountable for all aspects of the work in ensuring that questions related to the accuracy or integrity of the work are appropriately investigated and resolved.

## Data Availability

The analyzed datasets generated during the study are available from the corresponding author on reasonable request.
